# Influencing Change: When “Best Practice” Changes and the Prototypical Good Farmer Turns Bad

**DOI:** 10.3389/fvets.2020.00161

**Published:** 2020-03-31

**Authors:** Laura Green, Jasmeet Kaler, Nicola Liu, Eamonn Ferguson

**Affiliations:** ^1^Institute of Microbiology and Infection, College of Life and Environmental Sciences, University of Birmingham, Birmingham, United Kingdom; ^2^School of Veterinary Medicine and Science, University of Nottingham, Leicestershire, United Kingdom; ^3^School of Life Sciences, University of Warwick, Coventry, United Kingdom; ^4^School of Psychology, University of Nottingham, Nottingham, United Kingdom

**Keywords:** sheep, treatment of lameness, longitudinal intervention study, prototypical farmer, behavioral change, trust, cultural capital, cognitive dissonance

## Abstract

Twenty-nine farmers with a flock prevalence of lameness >5% were visited in 2013. They participated in a facilitated discussion on treatment of footrot, and evidence-based new “best practice.” One year later, farmers were revisited and management and motivators for change were discussed. Farmers were asked how they would persuade other farmers to adopt “best practice.” Initially, most participants were resigned to having lame sheep. They believed that prototypical “good farmers” (including trusted family) practiced foot trimming, the traditional “best practice” and that the new “best practice” would be expensive and time consuming. Between 2013 and 2014 lameness prevalence reduced from 7.6 to 4.3%. The major behavioral changes were reduction in foot trimming, increased use of antibacterials to treat footrot, and treating sheep within a week of becoming lame. In 2014, participants were re-interviewed. They reported that an increased knowledge of the evidence-base, trust in the facilitator and talking to other trusted farmers who had already adopted the new “best practice” overcame concerns about the prototypical “good farmer” and motivated change. Persistent change occurred because participants observed health benefits for their sheep and that the new “best practice” had saved time and money. Participants stated that other farmers would be convinced to change to the new “best practice” because it saved time and money, ironically, these were among the original barriers to change. This is possibly an example of cognitive dissonance because farmers had become positive about the benefits of saving time and money following a change in their own behaviors.

## Introduction

Footrot is one of the top five most important diseases of sheep globally. Footrot causes pain (1), which is expressed as lameness (2), and so reduces welfare. It also reduces productivity, notably in numbers of lambs reared and lamb growth rate in meat sheep (3, 4). In England in 2013, ~5% of sheep in the population of 16 million adults were lame at any one time, with ~70% of lameness caused by footrot (5). Individual sheep recover from footrot most rapidly when treated with parenteral and topical antibiotic without trimming the hoof horn (6, 7). That treatment, when administered within 3 days of onset of lameness (3, 5), is current “best practice” because it minimizes the adverse effects from footrot on the individual sheep (3), reduces repeat cases of disease (7) and onward spread of disease (8). Current “best practice” has been promoted since 2006, and by 2013 11% of English farmers were using the new “best practice” (9).

Current “best practice” is quite different from past “best practice” which for centuries included foot trimming and topical treatment (10). Foot trimming diseased sheep evolved to routine inspection of the whole flock with regular foot trimming and foot bathing of all sheep ideally twice per annum, to treat footrot and reshape overgrown feet. That practice was recommended by national agricultural advisory bodies from the 1970s and is still included in some literature as a part of good sheep welfare [(11)^1^]. Managing footrot is often first learned on the family farm from forefathers as tacit knowledge and became a cultural norm (12) that underpins perceptions of the ideal or prototypical “good farmer” (13) as one who practiced whole flock measures of foot trimming and foot bathing. The wider literature on behavior change shows that people may have a positive image of the ideal or prototypical person, as was the case for many years with the prototypical smoker, and their closeness to this prototype drives their behavior (14, 15). Once a positive image is linked to a behavior it is difficult to change. In 2007, the majority of 170 farmers who responded to a questionnaire reported that foot trimming and foot bathing were their ideal managements to control footrot, whilst also reporting that they were not effective, an example of cognitive dissonance (16). They considered that treatment of individual lame sheep was time consuming and costly [although most sheep farmers are not able to calculate their income and expenditure (17)] and therefore not ideal.

In 2013, a questionnaire was sent to 4,000 randomly selected English sheep farmers, ~35% replied (5). The questions covered farmers' management practices to treat and control footrot and farmers' knowledge, attitudes and beliefs about footrot. It also captured farmer personality using the big five traits (18). A high flock prevalence of lameness was associated with farmers with less knowledge of the etiology of footrot, and of current “best practice,” and those that expressed negative emotions of anger and misery, and feelings of helplessness. Farmers who scored higher in trait conscientiousness and understood the importance of proactive control of lameness had a lower mean flock prevalence of lameness. As highlighted above, only 11% of farmers were using best practice and they had a mean <2% flock prevalence of lameness (9).

Much of the explanation for the high flock prevalence of lameness in Winter et al. (5) was associated with farmers still following past “best practice.” This included continuing to trim hoof horn to expose diseased tissue (therapeutic foot trimming) without use of an antibiotic injection to treat footrot. In addition, many farmers did not treat lame sheep within 3 days of onset of lameness, explained in part by farmers not prioritizing lameness all year round and not treating lame sheep at all during the mating season and late pregnancy (9). O'Kane et al. (9) proposed that feelings of hopelessness reported by farmers could trigger inactivity in managing lameness and create a cycle of self-fulfilling behavior, and that farmers' ability to act appropriately toward footrot was predicted in part by their perceived behavioral control, this has been reported for other animal diseases (19, 20).

In 2014, 32 compliant farmers from the 2013 respondents, with >300 ewes and >5% prevalence of lameness (with <3% of lameness caused by contagious ovine digital dermatitis, another infectious cause of lameness) were enrolled in a clinical trial. The intervention started with a visit from the research team who facilitated a one-to-one discussion around the use of current “best practice” on that farm. One year later, 29/32 farmers completed a second questionnaire. The geometric mean period prevalence of lameness changed significantly (*P* < 0.05) between 2013 and 2014 from 7.6% (7.1–8.2%) to 4.3% (3.6–5.0%), with 28/29 flocks reporting a reduction in prevalence of lameness. The key behavioral changes were a reduction in therapeutic and routine foot trimming and an increase in negative attitudes toward foot trimming, an increased use of parenteral antibiotics to treat footrot, and slightly more rapid time to treatment of lame sheep, all aspects of current “best practice” (21).

The one-to-one trial led to greater absolute and relative reductions in the flock prevalence of lameness than other parts of the trial that tested the impact of group discussions and individual farmers contacted by post with a leaflet [Supplementary Figure 1; (21)]. The benefits of one-to-one facilitated discussions include the opportunity to justify and explain information, overcome myths and misconceptions and increase a sense of control and self-efficacy in the learner (22, 23). These should have positive effects on mood, cognition (beliefs) and behavior (24). Researchers have proposed that accepting new information also depends on trust in the informant (25). Learners, here, the farmers, appraise their informant (the researcher) as well as the information that they are given. Trust can come from a positive belief in previous information from that individual or from trust in others in the same social category as the informant (25) or the learner (26).

In the current paper, we explore the barriers, mechanisms, and motivators of the 29 farmers who were interviewed twice, once in 2013, when they engaged in a facilitated discussion on best practice treatment of footrot, and again in 2014, when they reflected on changes in management of lameness in their flock over the 12-month period and their perception of what they had changed, why, and their motivation to continue with their new “best practice.”

## Materials and Methods

### Ethical Considerations and Design of the Intervention

Ethical approval for this study was granted in 2012 by the University of Warwick Biomedical and Scientific Research Ethics Committee (BSREC 159-01-2012).

The research team (LG, EF, JK, and Amy KilBride) met with two health psychologists (Ronan O'Carroll and Charles Abraham) expert in intervention study design for a discussion on the content of the intervention. A summary of the evidence for the most effective management of footrot, together with information on sheep farmers' attitudes to footrot (3, 6, 16) were presented and discussed. The experts proposed using reduced income as a motivation to change farmers' behavior. It was agreed that this was potentially a motivator for change but the research team highlighted evidence that the economics of sheep farming were dependent on subsidies and were highly volatile and not clearly linked to improved management, partly because few sheep farmers kept production records (17). One focus of the discussion was around changing farmer behavior on foot trimming, which they would have practiced for many years. One expert highlighted that foot trimming could be considered as a placebo—that because it was invasive it “must” be effective, it is reported that severe placebos are considered more effective than mild placebos (27). In the current study the placebo would be the farmer's view of efficacy of foot trimming on recovery from footrot. Should farmers view foot trimming in this way then changing behavior would require considerable evidence and possibly another activity to replace trimming.

Based on this discussion, a leaflet (Supplementary Figure 1) and talk with the intervention material were produced. The material included detailed explanation of why foot trimming was harmful whilst parenteral antibiotic was effective and also how to inject sheep with antibiotic. The latter was to encourage farmers to have an alternative activity to the foot trimming placebo and for education, because farmers' injection technique is often sub-optimal.

### The Interviews and Feedback Forms

In 2013, 32 sheep farmers with >300 ewes, who were not using current “best practice” to treat footrot, were identified from the respondents to the 2013 questionnaire (5). These farmers reported an average of >5% lame sheep in their flock and were not catching sheep within 3 days of onset of lameness. Farmers were visited in the summer of 2013; all participants were interviewed by Laura Green (LG). Jasmeet Kaler (JK) was present at the first 18 interviews to assess consistency of interviews and examination of sheep. Interviews lasted 15–75 min (median = 36 min).

**Figure 1:**
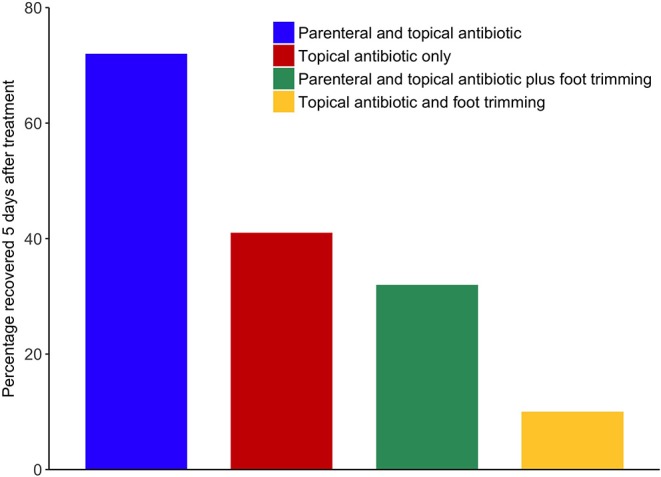
Bar chart on percentage of sheep recovered from footrot 5 days after treatment by treatment type, shown to farmers during the 2013 facilitated discussion. Adapted from article Green and Clifton (52).

In 2013, participants were asked to describe their farm and how they currently managed lame sheep. LG then initiated a facilitated discussion of current best practice to minimize lameness in their flock and whether and how the farmer might be able to adopt the new “best practice.” There were six key recommendations. These were to treat all sheep that became lame within 3 days; to treat all sheep with interdigital dermatitis or severe footrot (the two clinical presentations of footrot) with parenteral and topical antibiotics; to record all treatments electronically or on paper; to cull sheep lame with footrot twice or more in a year; and to stop practicing therapeutic or routine foot trimming. See Clifton and Green (52) for a summary of clinical presentations and recommended management practices and original evidence in Kaler and Green (53); Kaler et al. (7); Wassink et al. (3, 6), and Winter et al. (5). As part of the discussion, participants were shown a graph of recovery rates for four treatments for footrot to illustrate the benefits of using parenteral and topical antibiotics to treat footrot and the adverse effects of foot trimming on rate of recovery (Figure 1).

Within 2 weeks of the interview in 2013, all participants were sent the information leaflet (Supplementary Figure 1), and a feedback form, listing their specific recommendations given during the interview. Participants were asked to indicate whether they “will do,” “might do,” or “won't do” each recommendation and to return the feedback form to the research team. Participants were also asked how convinced they were by the interview using a five-point Likert scale ranging from “not at all convincing” to “very convincing.” The feedback form included an “other comments” section to allow for free text.

In 2014, the same participants were contacted to participate in a follow up interview. Twenty-nine farmers agreed to participate. Holly O'Kane (HO), who was not informed of discussions at the 2013 visits and had no contact with the farmers conducted follow-up visits in 2014; farmers were interviewed using a semi-structured approach. Participants were asked to recall the advice that they were given in 2013 and to discuss their motivations for, and barriers to, changing their management of lameness and adopting the recommended practices. Participants were also asked how other sheep farmers could be encouraged to adopt new “best practice.” Immediately after the second interview, participants were sent the 2014 questionnaire (21) on prevalence of lameness and managements used since the 2013 questionnaire; all 29 participants responded. All interviews from 2013 and 2014 were recorded and transcribed by Penguin Transcription, Watton, UK.

### Data Analysis

The 2013 and 2014 interview transcripts were imported and coded using the qualitative data analysis software NVivo 10 (QSR International). Ten transcripts (5 from 2013 and 5 from 2014) were selected at random, read and coded separately by two researchers (Nicola Liu (NL) and Claire Grant (CG) using thematic analysis (28). A coding scheme was developed and the qualitative data were categorized into particular themes and sub-themes (29). Themes and sub-themes were then compared between the two researchers to establish consistency, and that concepts and relations could be confirmed. The whole research team then reviewed the themes and sub-themes. The remaining 19 transcripts were then coded by NL. No new themes were identified from transcripts after the first 20 interviews, so data saturation was reached. Data from feedback forms and questionnaires (2013 and 2014) were also used in the analysis.

## Results

### Characteristics of the Farms and Participants

There were 32 farmers who participated in the interviews in 2013 and 29 farmers in 2014; the mean flock size was 821 (range: 320–2,600). The geometric mean prevalence of lameness was 7.6% [95% Confidence Interval (CI) 7.1–8.2%] and 4.3% (95% CI 3.6–5.0%) in 2013 and 2014, respectively (21). Of the 23 farmers that described their farming business, four only had sheep and 19 farmers also had other enterprises which included one or more of an agricultural business, arable, cattle or pigs. Participants had farmed sheep for 5–60 years (median 30) in 2013. The median percentage of working time that participants spent with their flock was 50% (range 10–100%, mode 90%).

### Themes That Emerged From the 2013 Interviews

Four themes emerged from qualitative analysis of the 2013 interviews, these were: (1) understanding the etiology and management of footrot, (2) barriers toward adopting best practice, (3) trust and knowledge transfer, and (4) attitudes toward scientific evidence.

#### Understanding the Etiology and Management of Footrot

Some farmers understood that footrot was an infectious disease; they explained that it could spread and a few participants noted that footrot is caused by a pathogen. In addition, most participants knew that antibiotic treatment was effective to treat footrot and stop its spread as described in the quote below.

“*These bacteria can grow at quite an alarming rate…We've had some awful weather…when one has it (footrot), the lot of them have it, so I try to inject and spray them with antibiotics before it gets out of hand”*

A few participants did not consider that interdigital dermatitis (scald) could progress to severe footrot nor that these were both presentation of footrot. This led to sheep with interdigital dermatitis being left untreated.

“*…if she's got only scald or an injury but she still feeding, it ain't such a big problem…I treat the ones that have [severe] footrot though. I inject them with antibiotics to kill off bacteria, but sometimes I don't feel they work very well”*.

Although the majority of participants sometimes used parenteral antibiotics to treat severe footrot, only a few used the correct dose based on weight; some were surprised to hear that they did not use the correct dose.

“*Participant: Oh really? We're not giving enough you see. We're giving 6ml…they are like 80 kilos (referring to the weight of their ewes). Interviewer: I think you'll find a dramatic difference if you go up to…”*

In addition, the duration between the first and the second dose of antibiotic injections varied considerably.

“*We jab them and then leave them until next time we bring in all the sheep…could be in 6 to 8 weeks, depends how busy we are. But anything that needs it then, it will get jabbed.”*

“*If she had [severe] footrot, I'd inject her with long-acting Terramycin. If she needed another, I'd inject her again in a week or two.”*

There was variability between whether participants treated groups or individual sheep as well as how quickly they treated (a) diseased sheep.

“*We tend to operate on individual treatment, any of the lame ones get treated within a week. Lame ones will get injected with antibiotics and the lame foot will get sprayed with Lincospectin as well.”*

“*When we see a lame sheep, we tend to wait until there is a group of them before we bring them in to treat them with antibiotic injection…or when it's time to footbath the whole flock.”*

Some participants marked their sheep after treatment, however, a couple of participants did not mark or record sheep that were treated because they believed they could recognize them by sight.

“*I don't mark ‘em…You won't believe it but certain sheep have certain, just have the appearance and you never forget the buggers…and when you've lambed ‘em and let them out in a field you know which lamb's off what…I know my sheep.”*

#### Barriers Toward Adopting Best Practice

Participants reported that factors such as economics of sheep farming, external pressures experienced from the media and their knowledge and belief about a certain treatment influenced their decision on whether to adopt recommendations provided by a sheep specialist. Overall, participants expressed strong emotions of frustration and defeat, these are contextualized and described below.

##### Cultural capital

Many of the participants reported that what they were taught at college or by their forefathers influenced their behaviors, such as continuing to practice foot trimming.

“*I have to trim, it looks messy otherwise…it's a habit I don't think I will be able to shake off…I mean you were taught to trim and I've been doing this for decades…both my father and grandfather did it.”*

“*Yeah, you were taught at college to do it [trim feet]…this was decades ago but it's still engrained in me. I don't know whether they still teach the young'uns that or not.”*

A few participants believed that farmers that foot trimmed sheep were “good farmers” and those who did not foot trim were “bad farmers” because a lack of action showed a lack of care.

“*Well, farmers who don't trim are thought of…as bad farmers and farmers that trim their sheep regularly are seen to be good farmers because they tend to their sheep more.”*

Farmers considered use (appropriate use) of antibiotic posed a threat to farmers “good farming' habitus and the quotes below illustrate their fears.

“*…we're frightened of it [antibiotics] escaping and getting into the food chain so that's one of the reasons why we don't really inject any lambs or ewes for footrot or anything.”*

“*the thing that plays on my mind is injecting too much antibiotics or too many times, again, throws up the problem similar with humans, the problem at the moment with getting immune to antibiotics that you hear on the news.”*

##### Economics

All participants mentioned economics as a major driver of decisions on whether to implement changes to their farming system. This delayed changes which would result in better control of footrot. The costs included treatment, management, labor, and veterinary services.

“*If things don't improve in the sheep industry, we might have to cut back on treatments and things, wouldn't be able to afford to carry on…I had a bill now back from my vet…one month from the guy who supplies me with the medicines and the Footvax and the sprays, -£3500, you think, god!…And that wouldn't be all of it, there's the vet costs on top of that.”*

The majority of participants considered treating sheep within 3 days of seeing them lame costly, time-consuming and incurring extra work. Many farmers considered that they would need to purchase mobile handling equipment to catch sheep which was considered prohibitively expensive. In addition, there was a feeling that there were limited actions that farmers could take, because of other tasks or responsibilities. Sometimes this was framed as a genuine case of time poverty, prioritizing other issues at certain times of year.

“*It's a shame really, it really is a time thing…we've got 300 acres arable so there's time when I'm busy with that and I just can't do it really. There's only so much I can do in a day or in a week or so, you know.”*

“*We've got 6,000 sheep about in probably 30 flocks; if you think it takes 20 minutes a flock to check ‘em, I mean that's 10 hours a day. So, two of us…if you were just to treat, you'd be chasing sheep all day long doing the lame ones.”*

A few participants had reduced the amount of hired labor to manage their flock to reduce costs. This might result in less attention for diseased and weak animals, which in turn, might increase the risk of disease transmission within the flock.

“*Some of the studies will say, oh it's worthwhile perhaps employing a shepherd to come in a day to do it, but it doesn't get gone and that's extra labour cost…we already had to let one of our shepherds go ‘cause we didn't have the money.”*

Most participants were reluctant to cull repeatedly lame sheep to prevent infection from spreading. Most farmers said they would rather persevere than cull sheep due to lameness, for fear of losing profit.

“*A ewe costs me £130 quid…if it had a poor foot we wouldn't mark it as a cull, no…'cause we can normally remedy a poor foot each time it's bad and it breaks my heart buying a ewe for £130 quid and then culling it for £60 the following year because it's got a poor foot.”*

#### Trust in Advice

In 2013 farmers placed value on social relationships, family and peers.

“*Yeah, we frequently speak to other farmers and see if they have any advice…we often talk to our vets too, they're very knowledgeable.”*

Some of the participants were guided by what their vets advised them in relation to whether to undertake certain treatments.

“*Years ago, I mainly used a footbath but for quite a few years now I've used the Terramycin spray because I didn't find footbathing very effective, and after talking to my veterinary surgeon he advised me to use this for footrot control. I've found it to be a great improvement…So I get advice from my vets and they are very helpful.”*

Two participants did not think their vets were experts and had very little contact with their vet unless they had e.g., a compulsory visit for bovine tuberculosis testing in their cattle.

“*I don't think they're experts, so I don't call a vet to come over unless…TB testing. I don't know, maybe they need more exposure to sheep farming so they know…better advice.”*

#### Attitudes Toward Scientific Evidence

A few farmers believed that foot trimming helped heal a foot with footrot based on their personal beliefs rather than scientific evidence.

“*No, you've got to trim. If she's lame and if a foot needs trimming back we cut it back quite hard, sometimes making it bleed to get the bad stuff out and let the air in to dry it quicker…I still feel that letting air in helps heal her.”*

All participants were shown a graph (Figure 1) with results from a clinical trial which highlighted the proportion of sheep recovered within 5 days after different treatments for footrot, the majority of farmers were positively influenced to consider the new approach to treatment by the evidence presented, the following quote is typical.

“*That's interesting. I didn't think trimming would slow recovery. And yeah, if you are saying, well you've proven that it targets at the disease quicker with antibiotics and…Yeah, I'd be quite happy to stop trimming them if it means that they will recover faster with antibiotics.”*

Overall, in 2013, there was a feeling of the inevitability of footrot, it is part of the life of a sheep farmer. However, this did not always lead to helplessness and defeat and, at least for one farmer, it was motivation to change.

“*…anyone who has sheep will have to deal with it [footrot]. There's no escaping that reality and sometimes there's nothing you can do ‘cause it's reached the point where it's damaged beyond repair…so we may try some of your advice.”*

In conclusion, the tone of the farmers on the topic of lameness during the 2013 interviews was generally negative with a belief that where there are sheep, there will always be lame sheep. Farmers' barriers were focused on concerns about the time and financial investment, the practicality of prompt treatment, justifiable use of antibiotics and change in the traditional way to manage lameness in sheep.

### Feedback Forms: Intention to Adopt the Recommended Advice

The participants' intention to change to the recommended advice was captured on feedback forms sent after the interview in 2013; 24/32 farmers returned these forms. Participants were given 5–8 recommendations in total, 25/26 participants were recommended to “treat lame sheep within 3 days” or “treat a third of the flock each day.” There were 5 (19%) participants who thought the information in the interview was very convincing, 19 (73%) thought it was convincing and the remaining 2 did not answer the question. At least two participants stated that they had already implemented changes before receiving the feedback form but other participants voiced concern about implementing certain recommendations. Examples of responses are below.

“*We have implemented the antibiotic route and markings and it seems to work 80% of the time. We will continue.”*

“*treating within three days is completely impractical and if not done constantly would not be worthwhile.”*

### Follow-Up Interviews in 2014

There were 29 farmers who were interviewed again in 2014. The prevalence of lameness had fallen in 28/29 flocks by a mean of 60% (21). In general, farmers' responses on intention to change in the feedback form was not the same as whether farmers changed their behavior in 2014. There were 5, 9 and 8 participants who stated that they “will,” “might,” and “won't” change to treat sheep within 3 days of becoming lame, in fact 1, 4, and 1 farmer respectively changed to treat sheep within 3 days and 4, 5, 7 farmers, respectively, to within 1 week (Table 1). Most farmers reduced/stopped foot trimming.

**Table 1:** Participants intention to adopt the recommendation “treat lame sheep within 3 days” or “treat a third of the flock each day” in 2013 by actual behavioral change in 2014.

**Farmer intention to change, 2013**	**Participants' actual change in behavior by 2014**			
	**Continued to treat in 3 days**	**Changed to treat in 3 days**	**Changed to treat in <1 week**	**Did not respond to 2014 questionnaire**
Will	1^*^	1	4	
Might		4	5	2
Won't		1	7	

* *1 farmer was treating lame sheep within 3 days and continued treating within 3 days of seeing lame sheep in 2014*.

Five themes emerged from the 2014 interviews, these were: (1) motivation to treat lame sheep and adopt new strategies, (2) resistance to change and facilitators of change, (3) rationalizing full and partial implementation, (4) changing behavior of others, and (5) changed attitudes toward managing lameness in 2014.

#### Motivation to Treat Lame Sheep and Adopt New Strategies

In 2014, all participants were asked their main motivation to reduce lameness since the 2013 interview. The most common response was to ensure that optimum sheep health and welfare was maintained; it was also considered a professional and moral obligation. There was a general consensus that lameness was the most important cause of poor welfare in sheep on farms and not managing lameness would result in losing time and money and increase labor demands. These were all themes or sub-themes framed around barriers.

“*Well, my first one is probably welfare, it's my job to look after them as appropriate. If you've got stock that aren't in good health they're not going to perform…the treatment, the time as well of treatment and cost, my time is a cost so it's all cost driven…But, the higher the scanning percent, the more milk it [investing in treatment] brings you, the better and fatter lambs you have, so the bank balance looks better.”*

One participant said they decided to try very hard to implement the recommended practices because this approach would maximize the change of saving time or money.

“*If it saves me time or money, then I'm willing to give it a go. Plus, if I'm doing the study I might as well go the whole hog and try this out ‘cause you've got nothing to lose in a way and maybe long term it could be much easier for you, which it is now…saved time and sheep looking healthier…”*

#### Resistance to Change and Facilitators of Change

A few participants expressed resistance to change their habits but were convinced by LGs reasoning behind each recommendation provided, and were trying to change. Motivators included increased understanding of the infectious nature of footrot and increased knowledge of treatment, trust in other farmers' experience and the interviewer, and spread of footrot.

“*I was extremely sceptical about this no trimming because you were always taught: you trim the feet, you spray the feet. And when she said to cut back on trimming ‘cause it did more harm, then showed that graph* (Figure 1)*, I was a bit…well, I thought trimming was good for the foot. But it was enough to convince me to…so I didn't do it with those fat lambs that were from last year and they were fine without it…although I still find it difficult not to grab the clippers.”*

A few of the participants said that other farmers (trusted informants) and practicality influenced them to stop foot trimming.

“*…hearing it from some other people like our neighbours, saying you know, less trimming is better, we've actually stopped doing that now…I think they're better. And I used to have help doing the sheep. I couldn't physically do them all on my own, so it suited me…less hassle, less labour and you can use that time wisely elsewhere…”*

Participants' awareness of their management strategies changed as a result of the discussion in 2013, both because the consequences of certain managements were explained and because farmers thought the interviews were educational and this increased awareness facilitated change as suggested by the views below.

“*I used to trim them right back. The problem was that we used to get a lot of them strawberry foot [granulomas], we don't get it much anymore. But I was told by the professor that it was ‘cause of me trimming too hard, and now I see it a lot less ‘cause I rarely trim.”*

“*the biggest benefit is being educated on how I can reduce the lameness…trying to treat them as soon as you see it ‘cause you think, oh it'll be alright, but they don't, once they've got it it stays; you have to try and treat it straight away and not leave it or else it spreads. But when you're busy doing other things, you don't always stop to think that way, so this study made me think differently.”*

#### Rationalizing Full and Partial Implementation: “Lower, Wealthier, Faster”

There were three main perceived benefits from the new “best practice,” lower prevalence of lameness, greater wealth and faster rate of recovery, which positively influenced participants' attitudes toward the recommended practices. Farmers reported that these benefits meant that the new practices were more likely to be continued after the study.

“*Definitely less work, healthier sheep, yeah. Less use of antibiotics so less cost ‘cause less are lame which is great…less use of your time and I think they recover faster if you jab ‘em and leave ‘em without trimming…but yeah I'm going to continue with this, it works.”*

Participants that were administering the incorrect dose of parenteral antibiotic found it easy to raise the dose and saw benefits rapidly and a couple of participants changed their culling policy. Again, these practices were more likely to be continued when the participants perceived the benefits.

“*Well, we've increased the dosage ‘cause that was easy to do, just add an extra mil or two and we've been doing that since last year and we've increased on our culling so we've culled pretty heavily and I think that's kept our lameness down so we will continue with them.”*

Although the majority of farmers had noticed beneficial changes (mentioned above), they did not implement all the recommended practices. Many participants explained why they had decided that it was unnecessary or impractical to adopt one or more of the recommended practices to treat lameness. The majority stated that they were unable to adopt the “treat sheep within 3 days of seeing them lame” recommendation due to difficulty of implementation; the same barriers that the farmers had raised to adopting any of the new recommendations were mentioned, it was perceived that it was not justified because of the cost, was time-consuming and was not possible to implement consistently; therefore practicality, time, habit were still the main barriers to change. The emphasis here is the word “can't.”

“*I did try but it can't be done. I still find the catching sheep in three days, that's fine for somebody sat in an office, not fine for us out in a field…It's impractical, it's hassle and we would need to get a Prattley [a portable race and pen]. It's going to cost me too much money and time. Yeah, that's the bit I found a bit challenging and I find that you can only do what was advised on occasions at best.”*

One participant stopped the recommended practices when they did not observe benefits or perceived them to not be working. This was the only flock where the prevalence of lameness increased between 2013 and 2014, from 15 to 25%.

“*I've tried what Laura recommended, just inject them and spray them and it doesn't work, so we've stopped doing it that way. I'm sorry. We're back to trimming and spraying.”*

#### Changing Behavior of Others: Advertise the Real Benefits or Do It One-To-One

Participants were asked the best method to convince and encourage other sheep farmers to adopt best practice. There was a general consensus that advertising the benefits of adopting best practice with case studies was the best method of changing habit(s) and taking up new recommendations.

“*Advertise in like Farmers Weekly and that, I wouldn't say the internet is the best thing, a lot of farmers are old and don't go on the internet. Farmers tend to…if somebody puts in a statement, ‘Oh we've dropped from 10% to 3% lameness' it makes you read it really because you're intrigued to see how they've achieved it. But I think you've got to show ‘em…It's got to be shown to be a financial advantage…Financial will work better than welfare.”*

However, some participants acknowledged that changing the minds of sheep farmers that were categorized as the “older generation” would be challenging as they have always managed lame sheep one way for many years or are resistant to being told what to do. With, many suggested targeting the “younger generation of farmers.”

“*People tend to put barriers up once they feel that they're being judged or they're being advised or told to do something which they're not happy with…For most farmers, it's how they've been brought up doing, they've been doing it for decades, so I think you've got to target the younger generation of shepherds rather than the older generation.”*

Although the majority voiced their opinion that advertising best practice alongside successful case studies was the best method of encouraging sheep farmers to comply with recommended practices, one participant was strongly opinionated and stated that educating sheep farmers one-to-one would help improve or change the way they managed and treated lame sheep. The participant admitted that they had seen similar advice as recommended during the interview in magazines such as “Farmers Weekly,” but felt that one-to-one interviews were the best form of communicating the recommendations and so that approach would have more success in changing attitudes and belief.

“*There's similar advice to what you people do…Farmers Weekly, but sometimes it's left on the coffee table, it soon collects dust and you bin it without reading it…I don't know, personally I felt this study helped more ‘cause the professor explained stuff that made sense with them foot ulcers [granulomas] and stuff…it's just things that might not be in magazines or leaflets, or it might have been but I've thrown it away…I think you would do better in trying to convince others if you went out and spoke to them. It's a good time to ask questions…”*

#### Changed Attitudes Toward Managing Lameness in 2014: A Positive Change “I'm More on Top of It…”

The interviews in 2013 and 2014 allowed the tracking of opinions, feelings and the changes in management made for 1 year after the facilitated discussion. The tone of farmers on the topic of lameness during the 2013 interviews was generally negative with a belief that where there are sheep, there will always be lame sheep. However, the attitudes during the 2014 interviews toward lameness were more positive with phrases such as “on top of things,” “feeling more confident,” “under control.”

“*…Yes, I'm more on top of it at the moment, that's the thing. I think it's good, I do really think that your general ideas are very good. I was brought up with, yeah, if it's lame you turn it over and you hack it all off and you go off from there, but then it makes sense when you really think about it, yeah that's an open sore, int it? It can't put its foot on the floor ‘cause you've cut it!”*

“*I feel more confident with how I'm treating lame ones now, and we stopped foot trimming and it's been a big labour saver…I think overall, our lameness is under control…”*

## Discussion

A number of key issues emerge from our analyses that highlight the barriers that farmers perceive that prevent or delay change in behavior, the mechanisms that lead to change, and the motivators that sustain changes in behavior.

A key barrier that emerges is around the practice of stopping foot trimming. Farmers current behavior, or habitual approach to managing lame sheep, which is steeped in tradition, includes the perception that the “good farmer” practices foot trimming both as part of treatment and as a routine flock inspection once or twice a year. Foot trimming has evolved into a cultural norm embedded in cultural capital (12). Cultural capital includes signs of prestige and status and can be gained and exist in forms of knowledge and skills, traditions, and habits (30) and can either cause resistance to, or facilitate the adoption of, practices (31). This is very much akin to the idea of a norm of best practice forming part of cultural evolution (12, 32). In this instance, these reflect the *vertical transmission* of norms of a “best practice” from one generation to the next. Such normative structures and beliefs are difficult to overcome because they are cemented by trusted information passed from a trusted and respected individual in one generation to the next. In addition, foot trimming has become a practice that has “moral” normative influence, which also explains some of the resistance some farmers reported to stopping foot trimming (33).

Trust, as we detail later, which we know as a key element in sustained behavior and behavior change, is central to supporting these cross-generational norms. Thus, any change is likely to be slow, especially in the current context of close families and communities of sheep farmers where trust is high (9, 16).

The challenge of instigating change in the context of close farming families is further compounded by the fact that such cultural norms are underpinned by the perceptions of the “good,” “ideal,” or “prototypical” farmer. The wider literature on behavior change shows that people may have a positive image of the ideal or prototypical person, as was the case for many years with the prototypical smoker and their closeness to this prototype drives their behavior (14, 15). Once a positive image is linked to a behavior it is difficult to change. This perfect storm of foot trimming linked to a positive image of the prototypical good farmer and using antibiotics linked to the current image of the prototypical bad farmer, that is cemented by vertical intergenerational and horizontal transgenerational trust, makes stopping foot trimming and using more antibiotics hard barriers to overcome.

Some farmers were reluctant to use antibiotics, with concern about them “escaping” into the environment given the current discussions on spread of antimicrobial resistance. This belief highlights *horizontal transmission* of norms in terms of following a local leader (12). There is currently strong emphasis on reduction of use of antibiotics in farm animals (34, 35). In fact, the emphasis is to use antibiotics appropriately, and treatment of individual sheep with footrot, a bacterial disease, is an appropriate use [(36)^2^]. Farmers appeared to gain confidence in appropriate use of antibiotics, including correct dosing, as a result of Figure 1 and the discussion in 2013.

Following from the above we can consider the mechanisms that can be targeted to bring about change. Trust is one of these. Different sources of information attract different levels of trust (37, 38). Farmers trust family and peers and seek advice from them (9, 39, 40). One important source of trusted information comes from the prototypical farmer (see above). If this prototype is the trusted traditional farmer then rather than try to undermine this source, it would be better, for example, to build up trust in other competing sources. In this case, several farmers reflected that they trusted the facilitator (LG), a competing source of trust offering different advice from the social norms; some reflected that by agreeing to be in the trial they had acknowledged that trust. Trying to discredit an already trusted source may result in “reactance,” whereby people actively disengage with the new system, or even sabotage it, as they feel their freedom (perceived control) has been taken away (41). This might have occurred with the one farmer who was adamant that stopping trimming was not possible, despite the prevalence of lameness increasing on his farm.

There was a mixed perception by participants' of the perceived knowledge and trustworthiness of their veterinary practitioner, which might be explained by a farmer with regular contact with their veterinarian that increased levels of perceived knowledge and trustworthiness where the farmer was likely to seek advice from the informant (vet), whereas other participants did not trust their veterinarian and perceived lack of knowledge (42). In general, sheep farmers report that they trust their vet for advice (9, 16), however, this trust is generally limited to advice around management of diseases that farmers are not familiar with, and trust is lost when veterinarians are perceived to lack knowledge of sheep farming or when farmers do not have regular support from the same veterinarian (17).

Perceived control comes out in the farmers' narratives clearly. Perceived control is often linked to perceived action-outcome linkages and agency (43, 44). If the farmer feels that they are actively involved in the treatment (which is more likely to be the case with trimming) and they see a positive consequence of their actions, they are more likely to find it personally beneficial and enact it in the future (44, 45). Thus, interventions that make the action (treating with antibiotics rather than trimming)—outcome (reduced lameness) more visible should be effective, and that is exactly what is observed in this study by the use of the graph (Figure 1). If this can be reinforced by (1) having farmers monitor their own flock more closely and ask them to note down when a treated sheep becomes well and (2) showing comparatively how much better antibiotic treatment is over foot trimming, this is likely to enhance coherence and cognitive participation both of which significantly impact adoption. We do not know whether the leaflet with careful description of how to administer and antibiotic injection was helpful to farmers or helped to remove the placebo belief that rigorous foot trimming must be good because the farmers were “doing” something not just “jabbing” and leaving the feet. No farmers mentioned the leaflet in their 2014 interview but many farmers reported receiving information on lameness in sheep from many sources that year (21).

Perceived control is also apparent in the farmers' descriptions of the inevitability and predictability of footrot. Predictability implies controllability; however, this can be paradoxical if you cannot control the outcome despite the predictability which can lead to depression and low mood (46), this was apparent in the 2013 interviews. If you know that something is definitely going to occur and you can put strategies in place to prevent it, this will not only treat the problem but enhance mood. However, this may also require a change in cognition about control. That is, if footrot is always there and foot trimming is therefore not preventing it, then maybe it is time to try something else: there is nothing to lose (47, 48). This idea was expressed by a few farmers and could form the basis of a novel intervention to change cognition and predictability and prevention. The wider literature shows that perceptions of control can increase through information provided, knowing there are alternative options, observing that others change and then enacting the behavior personally (43, 44).

Belief in self-control was highlighted as important in O'Kane et al. (9) where farmers who were more educated about lameness, and believed they had self-control, had a lower prevalence of lameness in their flock than farmers who did not believe they had self-control. In the current study we saw farmers develop a greater sense of self-control as their knowledge of how to treat footrot increased and their experience of the treatment being effective through the trial year. The farmers were clear that treating footrot reduced the prevalence of lameness in their flock resulted in healthier, “better looking” sheep.

The initial barriers to adopting the new behaviors (concerns about time and cost and tradition leading to difficulty in perceiving change in behavior) were not what influenced change. Farmers reported that they changed their behavior because they trusted the advisor, and they had agreed to be in the trial so felt a moral obligation to try the recommendations and, for many, they felt more informed with increased knowledge from the explanation of Figure 1.

In the follow-up interviews, moral beliefs about animal welfare were described as a motivator to use the new “best practice.” Care about animal welfare may reflect a self-presentation as a good person and may act as a signal to other farmers. If this is the case, then a focus on moral foundations about caring and harm minimization may offer a novel insight into interventions to help re-describe the ideal prototypical farmer. Moral beliefs and emotions may also be an interesting avenue for future research. Shame, for example, at not acting in a morally accepted way, is a strong motivating force to make *reparation*s and change behavior (49). If a moral norm is set up based on the new “best practice” as best for animal welfare then violating this norm may result in negative emotions that the farmer wishes to avoid and this shame-avoidance may well motivate sustained behavior change (49, 50).

While moral emotions—like shame—detailed above may be important mechanisms to motivate change, so are less internalized ones such as wealth generation making for wealthier farmers. Whilst farmers were skeptical of the new recommendations because they viewed them as likely to take up more time and cost money, after 1 year many reflected that the new “best practice” saved them time and money. Thus, there are multiple motivations that may be generating sustained behavior change, the avoidance of negative emotional states and generation of positive outcome such as financial benefits. These are often pitted against each other as different motivations to target, however, the farmers narratives suggest that these can be combined.

While participants were asked how they would influence other farmers to adopt the new “best practice” some farmers recommended farming magazines and case studies, whilst others said that these were rarely read and would be less effective than one to one communication. Both comments were valid. The farmers in the current trial were part of a larger trial where interventions were messaged by post, in small groups with a presentation plus question and answer session, and using the one to one face to face facilitation meeting. The intervention was increasingly successful as the contact with farmers was more personal (21). When asked what would convince other farmers to change, the participants overwhelmingly said that the key message was that new “best practice” saved time and money. Ironically, these were the two strongest barriers to change raised by the farmers at the start of the trial and they were not the motivators that farmers reported which were trust and increased knowledge about the best treatment for footrot. This could be an example of cognitive dissonance (51) because the farmers have changed their behavior and are now so positive about the benefits of saving time and money that they no longer recall that these were their own perceived barriers to change.

## Conclusions

We conclude that one-to-one facilitation including scientific evidence provided by a trusted advisor was reasonably successful in influencing farmers to change their treatment of footrot in sheep. The current paper together with previous work indicate that these approaches are probably robust in many situations, although not without substantial cost. That the barriers to change, time and money, were not the motivators for change. We conclude that it is therefore important when a programme of work to bring about change in practice is proposed, that the motivators for change are identified before the programme commences and used as the focus of influence. Motivators for change need to be elucidated carefully since at the end of the trial the farmers reflected the benefits of change that they observed, a healthy flock, time, and money saved, as potential motivators for other farmers, whereas these were the initial barriers for change.

## Data Availability Statement

The datasets generated for this study are not available due to confidentiality agreements with the farmers. Requests to access the dataset can be directed to the corresponding author.

## Ethics Statement

The studies involving human participants were reviewed and approved by ethical approval for this study was granted in 2012 by the University of Warwick Biomedical and Scientific Research Ethics Committee (BSREC 159-01-2012). The patients/participants provided their written informed consent to participate in this study.

## Author Contributions

LG, EF, and JK designed the study with input from Amy KilBride. LG managed the farmer interviews with assistance from JK in 2013 and Holly O'Kane conducted and organized the interviews in 2014. NL conducted the thematic analysis with input from LG, EF, and JK. LG, EF, and NL drafted the manuscript. JK contributed to interpretation of the data. All authors contributed to the manuscript editing and approved the final manuscript.

### Conflict of Interest

The authors declare that the research was conducted in the absence of any commercial or financial relationships that could be construed as a potential conflict of interest.

## Footnotes

^1^https://assets.publishing.service.gov.uk/government/uploads/system/uploads/attachment_data/file/69365/pb5162-sheep-041028.pdf

^2^https://www.farmantibiotics.org/wp-content/uploads/2017/06/Antibiotics-and-lameness-in-sheep.pdf
