# Supplementary material for: Influencing Change: When “Best Practice” Changes and the Prototypical Good Farmer Turns Bad
Source: Front Vet Sci. 2020 Mar 31;7:161. doi: 10.3389/fvets.2020.00161 (PMC7136422; doi:10.3389/fvets.2020.00161)

Frequently asked questions

How is this new approach different from previous management?
The new approach is to treat all ewes (not some) with scald and footrot with antibiotic injection and spray (with no foot trimming) within three days (no longer) of first becoming lame. This leads to the fastest recovery from lameness and reduces spread of footrot and scald.

1. CATCH within three days

Why should I treat within 3 days? Footrot and scald spread from sheep to sheep so treating lame sheep quickly stops infection spreading and avoids ewes losing condition and not feeding their lambs.

Isn't catching individual sheep going to take me a lot of time? Each sheep farmer has their own way of catching sheep, you are the experts. Farmers say they find it quicker to catch and treat individual lame sheep than gather sheep for a whole group treatment. Once you have footrot and scald under control (6 – 8 weeks) you will have very few / no lame sheep to catch at each check.

What if I don't want to catch ewes with young lambs? You will get very few lame ewes with lambs in the main group if you only turn sound ewes with lambs into this group. Keep lame ewes (even once treated) with lambs in a separate group.

2. INSPECT the feet, do not trim

Why should I never trim feet when sheep have scald or footrot? New research shows trimming hoof horn delays healing and increases the chance that sheep will get footrot again.

Don't I need to trim the hoof to let the air in to help it heal? Because the antibiotic injection works from inside the foot there is no need to expose the lesion to air, once the sheep is sound she will bear weight on the foot and wear away loose and overgrown horn.

3. DIAGNOSE the cause

How do I know it is footrot unless I trim the foot? You can recognise footrot by its smell, so you do not need to trim the hoof horn. If the sheep has an abscess or shelly hoof you might need to trim the horn. If unsure of the cause of lameness consult your vet.

4. TREAT with antibiotics

Why should I ALWAYS use antibiotic injection? Because injected antibiotics reach bacteria deep in the foot that a spray or footbath cannot reach and so lead to rapid and better recovery.

Won't the bacteria become resistant to antibiotics? Antibiotics are the correct treatment because footrot and scald are caused by bacteria. Over a matter of weeks the number of cases go down when you manage as above, and you will use less antibiotic in the long run.

What about antibiotic withdrawal periods in my lambs? Injecting lambs with footrot will keep them growing: some antibiotics have a short withdrawal period; discuss this with your vet.

Why do I need to use the spray as well? Using a spray along with the injection speeds up healing and helps prevent footrot spreading.

How will the spray work if I don't trim the foot? Sprays clear the bacteria on the outside of the foot, the injection will treat bacteria deep in the foot.

Why should I treat heavily pregnant ewes? Clearly you need to be careful catching these ewes but the benefits are less pain for the ewe, better body condition, larger birth weight and better fed lambs.

What about other flock treatments, such as footbathing and vaccination? Footbathing can prevent footrot and scald, e.g. footbath sound sheep at housing. It can also be used to treat outbreaks of scald but not footrot. Farmers often find that they do not get scald outbreaks once following the new recommendations. Even when you vaccinate you will have some sheep with footrot and scald. Treat these within three days using antibiotic injection and spray all four feet.

Should I give lame sheep pain relief? It is best practice to give painkillers when animals are in pain, and lameness is painful. Ask your vet for details.

5. MARK and RECORD

What is the benefit of marking and recording sheep with footrot and scald? Marking and recording helps to identify the repeated cases of footrot and scald which should be considered for culling.

6. CULL repeat cases

Why should I cull sheep that have had footrot several times? These sheep spread footrot to other sheep. If you cull them you will reduce the amount of lameness in your flock and save time, effort and money on treatment.

Contact: Amy KilBride, School of Life Sciences, University of Warwick, CV4 7AL.
Tel: 02476 575 862 Email: a.kilbride@warwick.ac.uk

NEW recommendations for controlling footrot and scald

Don't miss out on the benefits for you and your flock

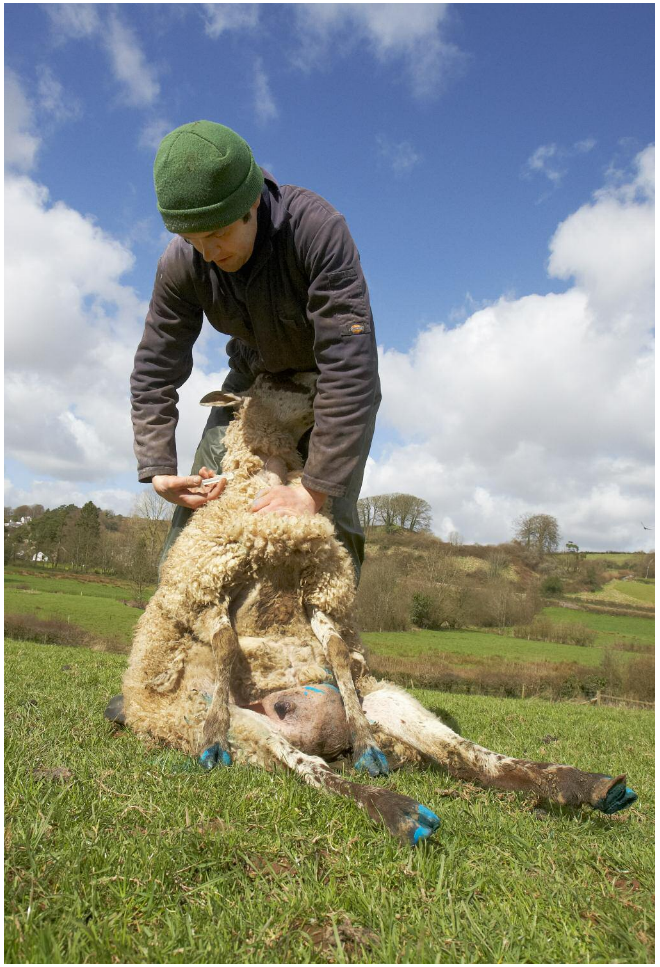

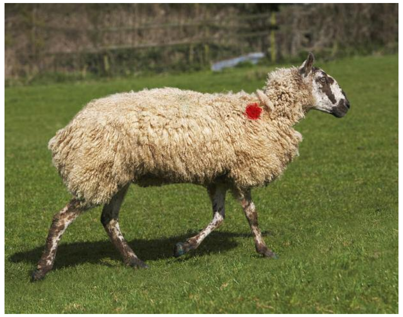

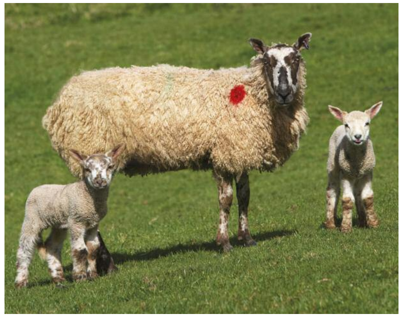

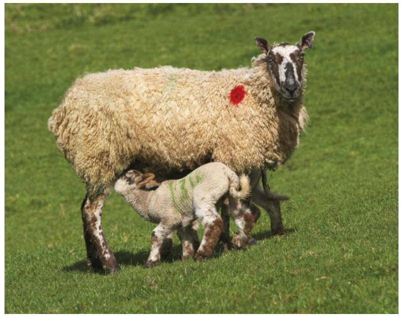

six steps to
sound sheep

We know that sheep farmers work tirelessly to control footrot and scald in their flock

Lameness costs £6 - £10 for every ewe tupped

Previous recommendations for management of lameness have been updated based on new research from the Universities of Warwick and Nottingham that involved over 1500 British sheep farmers. This new work shows that farmers can keep levels of lameness at less than 2% by treating all cases of footrot and scald using Six Steps:

Take **Six Steps to sound sheep**

1: **CATCH** sheep within **three** days of becoming lame

2: **INSPECT** the feet, clean away dirt, **do not trim** hoof horn

3: **DIAGNOSE** the cause of lameness

4: **TREAT** all **ewes and lambs with footrot** with an **antibiotic injection** and **spray** all four feet, **do not trim** hoof horn

TREAT all **ewes with scald** with an **antibiotic injection** and **spray** all four feet, **do not trim** hoof horn

TREAT all **lambs with scald** by **spraying** all four feet, **do not trim** hoof horn

5: **MARK and RECORD** all sheep with footrot or scald

6: **CULL** sheep that are repeatedly lame

“In my flock, lameness levels have dropped from 8% to 1% using these new recommendations. We continue to put time into keeping levels of lameness low but at least now we have a strategy to treat lame sheep that works.

Huw Davies, sheep farmer, Carmarthenshire

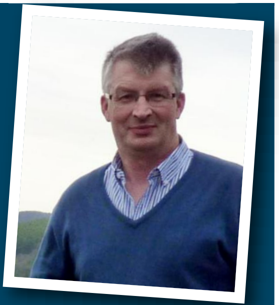

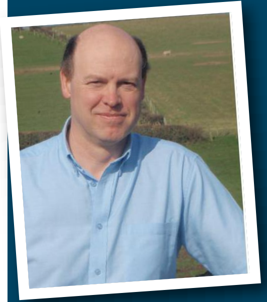

Six Steps is the standard approach I’ve used with my clients for the last four years. Treat sheep as soon as you see them lame, don’t wait for five or six to be lame, identify the cause, use an antibiotic spray and oxytetracycline injection and don’t trim. When you remember that footrot and scald are infectious diseases, it makes sense that antibiotics work and the foot does not need trimming.”

Iain Richards, sheep vet, Cumbria

Don’t miss out on the benefits for you and your flock

By not following this simple Six Steps programme, you will:

- continue to have high numbers of lame ewes in your flock
- continue to have a ewes that remain lame for more than a week
- continue to have ewes in poor body condition because they are lame for more than a week
- continue to have outbreaks of scald in ewes and lambs
- waste time with unnecessary foot trimming and footbathing
- continue to have lower production levels- low lambing percentage, reduced lamb survival, slow lamb growth rates - because of high levels of lame ewes

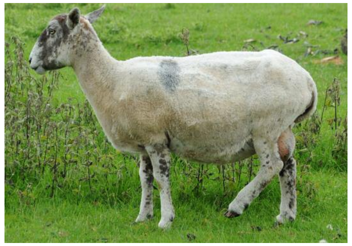

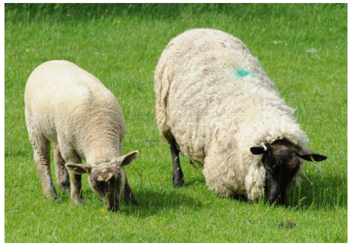

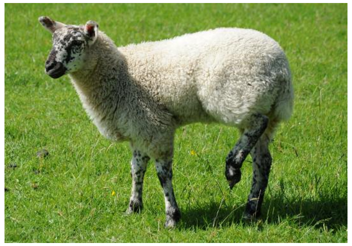

Supplement: Supplementary file 1 [file Image_1.pdf]
